# Plant versus animal based diets and insulin resistance, prediabetes and type 2 diabetes: the Rotterdam Study

**DOI:** 10.1007/s10654-018-0414-8

**Published:** 2018-06-08

**Authors:** Zhangling Chen, Maria Geertruida Zuurmond, Niels van der Schaft, Jana Nano, Hanneke Anna Hendrikje Wijnhoven, Mohammad Arfan Ikram, Oscar Horacio Franco, Trudy Voortman

**Affiliations:** 1 Department of Epidemiology, Erasmus University Medical Center, Office Na-2903, PO Box 2040, 3000 CA Rotterdam, The Netherlands; 2 Department of Health Sciences, Faculty of Earth and Life Sciences, Vrije Universiteit, Amsterdam, The Netherlands

**Keywords:** Cohort study, Epidemiology, Plant-based diet, Insulin resistance, Prediabetes, Type 2 diabetes

## Abstract

Vegan or vegetarian diets have been suggested to reduce type 2 diabetes (T2D) risk. However, not much is known on whether variation in the degree of having a plant-based versus animal-based diet may be beneficial for prevention of T2D. We aimed to investigate whether level of adherence to a diet high in plant-based foods and low in animal-based foods is associated with insulin resistance, prediabetes, and T2D. Our analysis included 6798 participants (62.7 ± 7.8 years) from the Rotterdam Study (RS), a prospective population-based cohort in the Netherlands. Dietary intake data were collected with food-frequency questionnaires at baseline of three sub-cohorts of RS (RS-I-1: 1989–1993, RS-II-1: 2000–2001, RS-III-1: 2006–2008). We constructed a continuous plant-based dietary index (range 0–92) assessing adherence to a plant-based versus animal-based diet. Insulin resistance at baseline and follow-up was assessed using homeostasis model assessment of insulin resistance (HOMA-IR). Prediabetes and T2D were collected from general practitioners’ records, pharmacies’ databases, and follow-up examinations in our research center until 2012. We used multivariable linear mixed models to examine association of the index with longitudinal HOMA-IR, and multivariable Cox proportional-hazards regression models to examine associations of the index with risk of prediabetes and T2D. During median 5.7, and 7.3 years of follow-up, we documented 928 prediabetes cases and 642 T2D cases. After adjusting for sociodemographic and lifestyle factors, a higher score on the plant-based dietary index was associated with lower insulin resistance (per 10 units higher score: β = −0.09; 95% CI: − 0.10; − 0.08), lower prediabetes risk (HR = 0.89; 95% CI: 0.81; 0.98), and lower T2D risk [HR = 0.82 (0.73; 0.92)]. After additional adjustment for BMI, associations attenuated and remained statistically significant for longitudinal insulin resistance [β = −0.05 (− 0.06; − 0.04)] and T2D risk [HR = 0.87 (0.79; 0.99)], but no longer for prediabetes risk [HR = 0.93 (0.85; 1.03)]. In conclusion, a more plant-based and less animal-based diet may lower risk of insulin resistance, prediabetes and T2D. These findings strengthen recent dietary recommendations to adopt a more plant-based diet.

*Clinical Trial Registry number and website* NTR6831, http://www.trialregister.nl/trialreg/admin/rctview.asp?TC=6831.

**Electronic supplementary material:**

The online version of this article (10.1007/s10654-018-0414-8) contains supplementary material, which is available to authorized users.

## Introduction

Diet is an important modifiable lifestyle determinant in the development of type 2 diabetes (T2D) [1]. Among these dietary determinants, several plant-based foods such as root vegetables, green leafy vegetables, whole grains, nuts and peanut butter, have been associated with a lower risk of T2D [2–5]. By contrast, several animal-based foods, including red meat, processed meat, and daily consumption of eggs have been associated with an increased risk of T2D [4, 6, 7].

Although multiple food groups seem to influence the risk of T2D, humans generally do not consume single food items or food groups, and the role of diet in health may be better described by overall dietary patterns [8]. Previous studies have observed that vegan or vegetarian diets are associated with improved glycemic control [9] and lower T2D risk [10]. However, these previous studies dichotomously classified participants, and only defined diets as vegetarian or vegan versus non-vegetarian diets. A dichotomous classification of vegans or vegetarians versus their non-vegetarian counterparts might not be an optimal approach in understanding the effect of a plant-based diet in Western countries, because it does not reflect dietary patterns of a large proportion of the population. For public health advice, it is interesting to know if a more plant-based and less animal-based diet may also influence insulin resistance and risk of prediabetes and T2D beyond strict adherence to a vegetarian or vegan diet. To our knowledge, only one previous study, a large prospective cohort study in the US, examined associations between variations in the degree of adherence to plant-based versus animal-based diets with T2D risk and observed that a more plant-based diet was associated with a lower T2D risk [11]. Studies on the associations of such plant-based dietary patterns with T2D risk in other populations are needed. In addition, the association of such plant-based dietary patterns with intermediate risk factors for T2D, such as insulin resistance and prediabetes remains unknown.

Therefore, we aimed to investigate whether adherence to a more plant-based, and less animal-based diet is associated with insulin resistance, and risk of prediabetes and T2D in a Dutch middle-aged and older general population.

## Methods

### Study design

This study was carried out within three sub-cohorts of the Rotterdam Study (RS), a prospective cohort study of adult aged 45 years and older living in the well-defined district of Ommoord in Rotterdam, the Netherlands. A detailed description of the Rotterdam Study methodology is described elsewhere [12]. Briefly, recruitment of participants for the first sub-cohort (RS-I) started in the period of 1989–1993 among inhabitants aged ≥ 55 years (n = 7983). In 2000–2001, the study was extended with a second sub-cohort (RS-II) of new individuals (n = 3011) who had become 55 years of age or moved into the study area after 1990. In 2006–2008, a third sub-cohort (RS-III) was recruited with new individuals aged 45 years and older (n = 3932). By the end of 2008, the overall study population contained 14,926 participants. Upon entering the study, participants underwent home interviews and a series of examinations in our research center every 3–5 years.

The Rotterdam Study has been approved by the institutional review board (Medical Ethics Committee) of Erasmus Medical Center and by the review board of The Netherlands Ministry of Health, Welfare and Sports. The approval has been renewed every 5 years. All participants gave informed consent.

### Population for current analyses

**Fig. 1:**
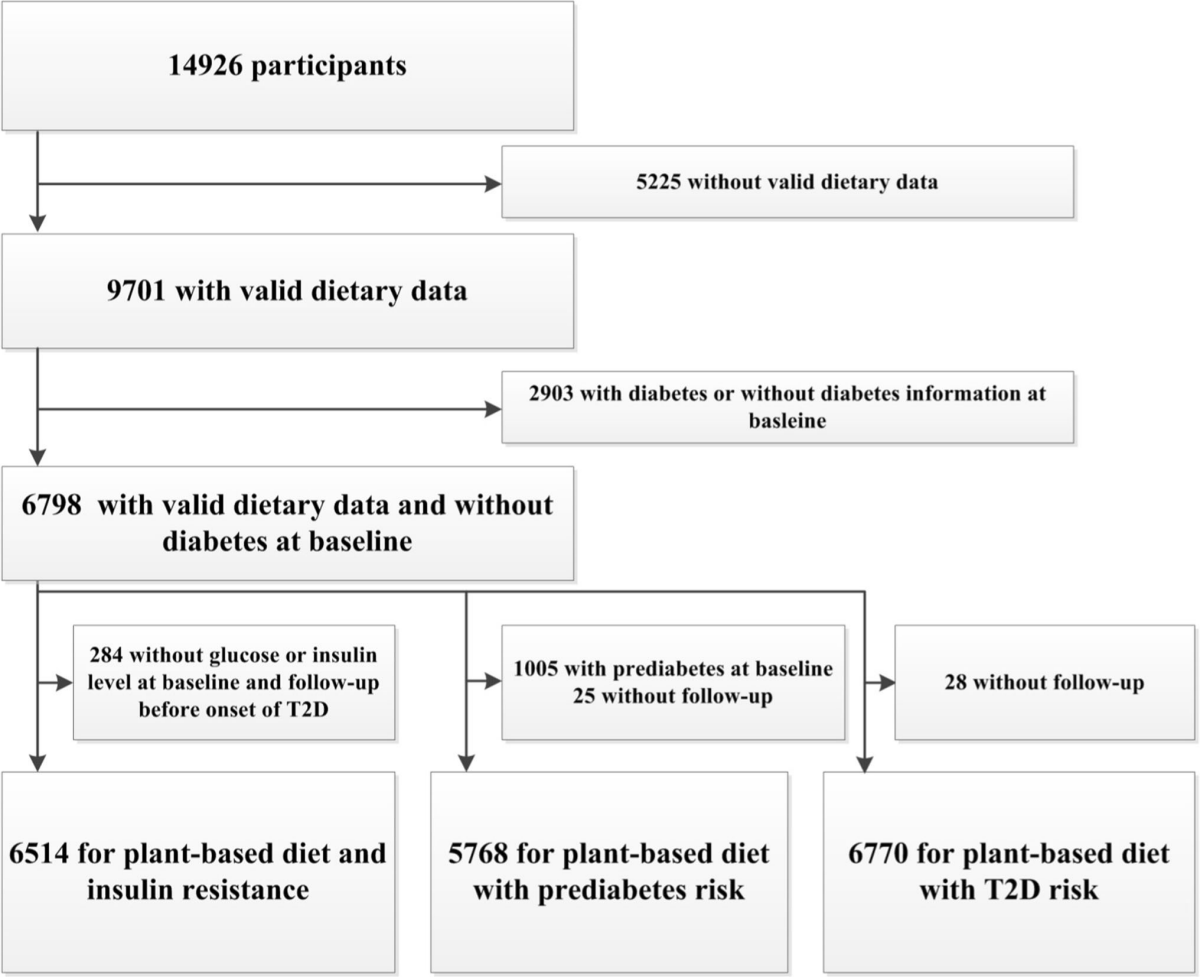
Flow diagram of participant selection

For the current study, we used data from all three sub-cohorts (Fig. 1). Of the 14,926 participants, we excluded those without valid dietary data (no dietary data (n = 5141) or unreliable dietary intake according to a trained nutritionist or an estimated energy intake of < 500 or > 5000 kcal/day (n = 84) [13]) at baseline (RS-I-1: 1989–1993, RS-II-1: 2000–2001, RS-III-1: 2006–2008), and those without diabetes information or with prevalent T2D at baseline (n = 2903), leaving 6798 participants included as main population for analysis.

From this group of 6798 participants, 6514 participants had data on HOMA-IR before onset of T2D and were included in the longitudinal HOMA-IR analyses. For the analyses on prediabetes risk, we excluded those with prevalent prediabetes at baseline (n = 1005) or without follow-up of prediabetes (n = 25), leaving 5768 participants. In the analyses assessing risk of T2D, we excluded participants without follow-up of T2D (n = 28), leaving 6770 participants. The flow-diagram of the included participants is presented in Fig. 1.

### Dietary assessment

Dietary intake was assessed at baseline in all three sub-cohorts using semi-quantitative food-frequency questionnaires (FFQ) as described in more detail elsewhere [13]. We used an FFQ with 170 food items to assess dietary intake at baseline of RS-I (1989–1993) and RS-II (2000–2001) [14]; and at baseline of RS-III (2006–2008) we used an FFQ with 389 food items [15]. The 170-item FFQ was validated in a subsample of the Rotterdam Study (n = 80) against fifteen 24-h food records and four 24 h urinary urea excretion samples [14]; and the 389-item FFQ was previously validated in other Dutch population against measurement of biomarkers, against a 9-day dietary record, and against a 4 week dietary history [16]. In general, the validation studies demonstrated that the FFQs were able to adequately rank participants according to their intake [13]. Food intake data were converted to energy and nutrient intake based on Dutch Food Composition tables (NEVO).

### Plant-based dietary index

We constructed an overall plant-based dietary index, which was a modified version of two previously created indices [11, 17]. More specifically, our index is similar to the “provegetarian food pattern” of Martínez-Gonzáles et al. [17] and to the “overall plant-based diet index” of Satija et al. [11], but was adapted to include slightly different types and numbers of food categories.

First, the food items as measured by the FFQs were divided into 23 food categories (Supplemental Table 1), on the basis of the main food groups in the Dutch diet and the Dutch food-based dietary guidelines [18, 19]. Twelve of the categories were plant-based and eleven were animal-based. Food items that were not clearly animal-based or plant-based, such as pizza, as well dietary supplements, were not included in the food categories for the index.

Dietary intake for each of the 23 food categories (g/day) was calculated for each participant. Subsequently, for each category, the intake was divided into cohort-specific quintiles. Each quintile was assigned a value between 0 and 4. For the twelve plant-based food categories, consumption within the highest quintile was scored a 4, consumption within the second highest quintile was scored a 3, and so on, ending with consumption within the lowest quintile receiving a score of 0. The eleven animal-based food categories were scored reversely: consumption within the highest quintile was scored a 0 consumption within the second highest quintile was scored a 1, ending with consumption within the lowest quintile receiving a score of 4. Furthermore, we ensured that all participants with null-consumption were given the score belonging to the lowest quintile by re-scoring when necessary.

Finally, these category quintile-scores were added up for per participant to create their overall score on the plant-based dietary index. The resulting index yielded a score for each participant that measured adherence to a plant-based versus animal-based diet on a continuous scale, with a lowest possible score of 0 (low adherence to a plant-based diet) and a highest possible score of 92 (high adherence: high plant-based and low animal-based). Information on intake of each food category across quintiles of scores on the plant-based dietary index is shown in Supplemental Table 2.

### Assessment of insulin resistance

Fasting blood samples were collected at RS-I (RS-I-3: 1997–1999, RS-I-5: 2009–2010), RS-II (RS-II-1: 2000–2001, RS-II-3: 2010–2011), and RS-III (RS-III-1: 2006–2008, RS-III-2: 2011–2012). Glucose levels were examined with the glucose hexokinase method. Serum insulin was measured by electro chemiluminescence immunoassay technology. Insulin resistance was calculated using the homeostasis model assessment of insulin resistance (HOMA-IR). The following formula was used: fasting insulin (mU/L) × fasting glucose (mmol/L)/22.5.

### Assessment of prediabetes and type 2 diabetes

Information on prediabetes and T2D was collected from general practitioners’ records, pharmacies’ databases, and follow-up examinations in our research center. Data of prediabetes and T2D in our analyses were collected until January 1, 2012. Prediabetes and T2D were identified according to WHO criteria: prediabetes was defined as a fasting blood glucose concentration of > 6.0 and < 7.0 mmol/L, or a non-fasting blood glucose concentration of > 7.7 mmol/L and < 11.1 mmol/L; T2D was defined as a fasting blood glucose concentration of ≥ 7.0 mmol/L, a non-fasting blood glucose concentration of ≥ 11.1 mmol/L (when fasting samples were unavailable), or the use of blood glucose-lowering drugs or dietary treatment and registration of the diagnosis diabetes. All possible cases of prediabetes and T2D were formally judged by two independently working study physicians or, in case of disagreement, by an endocrinologist [20].

### Assessment of covariates

Information on age, sex, smoking status, educational level, medication use, food supplement use, and family history of diabetes, was obtained from questionnaires at baseline. Information on physical activity was obtained using the adapted version of the Zutphen Physical Activity Questionnaire at RS-I-3 and RS-II-1, and using the LASA Physical Activity Questionnaire at RS-III-1. Physical activities were weighted according to intensity with Metabolic Equivalent of Task (MET), from the Compendium of Physical Activities version 2011. To account for differences between the two questionnaires, questionnaire-specific z-scores of MET-hours per week were calculated. At our research center at baseline, body weight was measured using a digital scale and body height was measured using a stadiometer, while participants wore light clothing and no shoes, and BMI was calculated (kg/m^2^). Information on hypertension, hypercholesterolemia, coronary heart disease (CHD), cancers, and stroke was obtained from general practitioners, pharmacies’ databases, Nationwide Medical Register, or follow-up examinations in our research center.

### Data analyses

To obtain a normal distribution for HOMA-IR, we applied a natural-log transformation. Non-linearity of associations of score on the plant-based dietary index with all outcomes were explored using natural cubic splines (degrees of freedom = 3). As no indications for non-linear associations for the main models were found, all primary analyses were performed using models assuming linearity. We examined the association between score on the plant-based dietary index with longitudinal HOMA-IR using linear mixed models, with a random-effects structure including a random intercept and slope (for time of repeated measurements of HOMA-IR). We examined the association between score on the plant-based dietary index and risk of prediabetes and risk of T2D using Cox proportional-hazards regressions. Hazard ratios (HRs) and regression coefficients (βs) were presented per 10 units higher score on the plant-based dietary index, along with the corresponding 95% confidence intervals (CIs). All analyses were performed in participants of the three sub-cohorts combined and in the three sub-cohorts separately.

All analyses were adjusted for energy intake, age, sex and RS sub-cohort in model 1, and for the analyses of longitudinal HOMA-IR we additionally adjusted for the time of repeated measurements of HOMA-IR. In model 2, we additionally adjusted for smoking status, educational level, physical activity, food supplement use, and family history of diabetes. Baseline BMI was added to model 3 to examine its potential mediating effect.

We examined effect modification by including interactions of the plant-based index with age, sex, or BMI for all outcomes in model 2.

Several sensitivity analyses were performed based on model 2. First, to check if the associations were driven by any specific components of the plant-based dietary index, we repeated our main analyses by excluding each one of the 23 components from the plant-based dietary index one by one at a time, and additionally adjusting for the excluded component. Second, to check if the associations were mainly driven by plant-based beverages combined, we examined the associations by excluding all plant-based beverages combined (category “coffee and tea”, category “alcoholic beverages”, and category “sugary beverages”) from the plant-based dietary index at a time, and additionally adjusting for them. Third, we examined the associations by excluding less healthy plant-based foods combined (category “sweets”, category “sugary beverages”, category “potatoes”, and category “refined grains”) from the plant-based dietary index at a time, and additionally adjusting for them. To further examine whether these less healthy plant foods contributed to the association of the plant-based dietary index; we created a less healthy plant foods score, for which, positive scores were given to these four types of less healthy plant-based food groups; and reverse scores were given to healthy plant food groups and animal food groups [21]. Fourth, to examine if potential associations of the plant-based dietary score with outcomes were independent of overall quality of the diet based on adherence to dietary guidelines, we examined the correlation between the plant-based dietary score and the dietary guidelines score; and we repeated analyses with additional adjustment for dietary guidelines score. Fifth, we additionally adjusted for hypertension and hypercholesterolemia. Sixth, we excluded the participants with chronic diseases at baseline, such as participants with CHD, cancers, or stroke, to exclude the possibility of a significant change of diet and life style at follow-up. Last, we excluded the participants who developed prediabetes and T2D in the first 2 years of follow-up in the analyses for risk of prediabetes and T2D, respectively.

Missing values on covariates (ranging from 0.3 to 3.9%) were accounted for using multiple imputations (n = 10 imputations). We used SPSS version 21 (IBM Corp., Armonk, NY, USA) and R version 3.1.2 (R Foundation for Statistical Computing, Vienna, Austria) to perform these analyses.

## Results

### Baseline characteristics

Baseline characteristics of the study population are shown in Table 1. In our population of 6798 participants, baseline scores on the plant-based dietary index (with a theoretical range from 0 to 92) ranged from 24 to 75, with a mean ± SD score of 49.3 ± 7.1. Mean age of the study population was 62.0 ± 7.8 years and 41.3% of the participants were male. Mean BMI was 26.6 ± 3.9 kg/m^2^. Characteristics were similar before and after multiple imputation (Supplemental Table 3). Supplemental Table 4 shows baseline characteristics of the participants not included in our analyses.

**Table 1:** Baseline characteristics of study participants (n = 6798)

Characteristics	Mean (SD) or %
Age (years)	62.0 (7.8)
Sex (% male)	41.3%
BMI (kg/m^2^)	26.6 (3.9)
Smoking status (%)	
Never	32.2%
Ever	45.1%
Current	22.7%
Physical activity^a^ (MET-hours/week)	
RS-I and RS-II (assessed with Zutphen Questionnaire, n = 4393)	86.7 (44.7)
RS-III (assessed with LASA Questionnaire, n = 2194)	58.4 (55.8)
Hypertension (%)	42.3%
Hypercholesterolemia (%)	45.4%
Family history of diabetes(%)	10.8%
Education level (%)	
Primary	11.8%
Lower	40.9%
Intermediate	29.0%
Higher	18.3%
Current food supplement use (%)	16.5%
Total energy intake (kcal/day)	2134 (615)
Plant-based food category intake^b^ (g/day)	
Fruit	212.2 (115.5; 332.3)
Vegetables	209.1 (147.9; 286.87)
Whole grains	105.7 (61.3; 152.5)
Nuts	3.9 (0.0; 12.0)
Legumes	4.1 (0.0; 19.4)
Potatoes	99.7 (61.4; 148.2)
Vegetable oils	19.7 (9.2; 30.0)
Tea and coffee	758.9 (580.4; 1000)
Sugary beverages	46.3 (0.0; 139.6)
Refined grains	50.7 (23.9; 102.1)
Sweets	63.8 (37.1; 97.4)
Alcoholic beverages	56.4 (4.9; 159.8)
Animal-based food category intake^2^ (g/day)	
Low-fat milk	82.3 (0.0; 232.3)
Full-fat milk	0.0 (0.0; 0.0)
Low-fat yoghurt	56.1 (0.0; 164.6)
Full-fat yoghurt	0.0 (0.0; 4.9)
Cheese	30.8 (20; 47.1)
Unprocessed lean meat	10.7 (4.3; 18.1)
Fish	15.9 (3.9; 30.7)
Eggs	14.3 (7.1; 19.6)
Animal fat	0.0 (0.0; 0.9)
Desserts/dairy with sugars	14.1 (0.0; 54.6)
Processed meat/red meat	86.8 (60.4; 118.9)
Plant-based dietary index (score)	49.3 (7.1)

Plant-based dietary index: a higher score indicates a higher adherence to a plant-based diet (theoretical range from 0 to 92). Values shown are based on pooled results of imputed data

*MET* metabolic equivalent of task, *SD* standard deviation

^a^Values shown for MET-hours are un-imputed; imputation was performed on z-scores of physical activity

^b^Variables expressed as median (IQR) because of their skewed distributions

### Plant-based dietary index and insulin resistance

After adjustment for confounders in model 2, a higher score on the plant-based dietary index was associated with lower longitudinal HOMA-IR [per 10 units higher score on the index: β = −0.09; (95% CI: − 0.10; − 0.08)] (Table 2). Adding BMI to the model (model 3), attenuated the association, but it remained statistically significant [β = −0.05; (− 0.06; − 0.04)].

**Table 2:** Associations of the plant-based dietary index with longitudinal insulin resistance (HOMA-IR), risk of prediabetes, and risk of type 2 diabetes

	β (95% CI) for HOMA-IR	HR (95% CI) for risk of prediabetes	HR (95% CI) for risk of type 2 diabetes
	n = 6514	n = 5768	n = 6770
Model 1	− 0.09 (− 0.10; − 0.08)***	0.88 (0.80; 0.97)**	0.82 (0.73; 0.92)***
Model 2	− 0.09 (− 0.10; − 0.08)***	0.89 (0.81; 0.98)*	0.82 (0.73; 0.92)**
Model 3	− 0.05 (− 0.06; − 0.04)***	0.93(0.85; 1.03)	0.87 (0.79; 0.99)*

Effect estimates are regression coefficients (β) for ln HOMA-IR or hazard ratios (HRs) for incidence of prediabetes or type 2 diabetes with their 95%-confidence intervals (95% CIs), per 10 units higher score on the plant-based dietary index. Estimates are based on pooled results of imputed data

Model 1 is adjusted for energy intake (kcal), sex (male or female), age (years) and RS sub-cohort (RS-I, -II, or -III); and only for the HOMA analyses additionally for the time measurements of longitudinal HOMA

Model 2 is additionally adjusted for education (primary, lower/intermediate, intermediate, or higher), smoking status (never, ever, current); family history of diabetes (yes, no, or unknown); physical activity (z-score of MET-hours/week); and food supplement use (yes or no)

Model 3 is additionally adjusted for BMI

*BMI* body mass index, *CI* confidence interval, *HR* hazard ratio, *MET* metabolic equivalent of task, *RS* Rotterdam-Study

*p < 0.05; **p < 0.01; ***p < 0.001

### Plant-based dietary index and incidence of prediabetes

During 43,773 person-years of follow-up amongst 5768 participants (median follow-up 5.7 years), 928 participants developed prediabetes. After adjustment for confounders in model 2 (Table 2), a higher score on the plant-based dietary index was associated with a lower incidence of prediabetes [per 10 units higher score on the index: HR = 0.89; (95% CI 0.81; 0.98)]. After additional adjustment for BMI (model 3) the association was attenuated, and no longer statistically significant [HR = 0.93 (0.85; 1.03)].

### Plant-based dietary index and incidence of type 2 diabetes

During 54,024 person-years of follow-up amongst 6770 participants (median follow-up 7.3 years), 642 participants developed T2D. In model 2, a higher score on the plant-based dietary index was associated with a lower incidence of T2D [per 10 units higher score on the index: HR = 0.82; (95% CI 0.73; 0.92)] (Table 2). Additional adjustment for BMI (model 3) attenuated this association, but it was still statistically significant [HR = 0.87 (0.79; 0.99)].

The associations between the plant-based dietary index with longitudinal insulin resistance, and risk of prediabetes and T2D were similar in three sub-cohorts (Supplemental Tables 5–7). Associations did not differ by age, sex or baseline BMI (p-values for all interaction terms were > 0.05).

### Sensitivity analyses

The exclusion of each one of 23 foods from the index one by one at a time did not substantially change the estimates (Supplemental Table 8). Excluding all plant-based beverages combined at a time (coffee and tea, alcoholic beverages and sugary beverages) did not substantially change the estimates [per 10 units higher score on the index, insulin resistance: β = −0.06 (− 0.10; − 0.03), prediabetes risk: HR = 0.93 (0.84; 1.02), and T2D risk: HR = 0.85 (0.80; 0.96)]. The estimates also remained similar after excluding these less healthy plant-based foods combined at a time (sweets, sugary beverages, potatoes, and refined grains) [per 10 units higher score on the index, insulin resistance: β = −0.09 (− 0.10; − 0.07), prediabetes risk: HR = 0.90 (0.84; 0.98), and T2D risk: HR = 0.83 (0.74; 0.94)], and the less healthy plant foods score was not associated with insulin resistance or with risk of prediabetes or type 2 diabetes [insulin resistance: β = −0.002 (− 0.01; 0.006), risk of prediabetes: HR = 1.00 (− 0.99; 1.01), and risk of type 2 diabetes: HR = 0.99 (0.98; 1.00)]. The Pearson’s correlation coefficient between the plant-based dietary score with the dietary guidelines score was 0.16 (*P* < 0.05); and controlling for the dietary guidelines score did not substantially affect the estimates [per 10 units higher score on the index, insulin resistance: β = −0.09 (− 0.10; − 0.08), prediabetes risk: HR = 0.91 (0.82; 1.00), and T2D risk: HR = 0.81 (0.71; 0.91)].

Additional adjustment for hypertension and hypercholesterolemia did not change effect estimates [per 10 units higher score on the index, insulin resistance: β = −0.08 (− 0.10; − 0.07), risk of prediabetes: HR = 0.90 (0.82; 0.99), and risk of T2D: HR = 0.84 (0.75; 0.94)], and estimates remained similar after excluding participants with chronic diseases at baseline [per 10 units higher score on the index, insulin resistance: β = −0.09 (− 0.11; − 0.07), prediabetes risk: HR = 0.88 (0.79; 0.97), and T2D risk: HR = 0.81 (0.72; 0.92)]. Finally, excluding participants who developed T2D or prediabetes in the first 2 years of follow-up modestly attenuated the associations for prediabetes [per 10 units higher score on the index, HR = 0.91 (0.83; 1.01)], and T2D [HR = 0.82 (0.73; 0.92)].

## Discussion

In this large population-based cohort, we observed that a diet higher in plant-based foods and lower in animal-based foods was associated with lower insulin resistance, and a lower risk of prediabetes and T2D, suggesting a protective role of a more plant-based opposed to a more animal-based diet in the development to T2D, beyond strict adherence to a vegetarian or vegan diet.

### Comparison with other studies

The inverse association between plant-based diets and T2D risk is in agreement with previous research showing lower T2D risk for vegans or vegetarians, compared to non-vegetarians [10]. Moreover, our observed associations confirmed the observations of Satija and colleagues in a US sample [11], the only other prospective study examining adherence to plant-based diets in a continuous graduation with risk of T2D. Compared to this previous study in the US population, we have extended this evidence by also showing associations between plant-based diets in a continuous graduation with earlier stages of the development of T2D: insulin resistance, and prediabetes in a European population.

Our results imply a beneficial effect of adherence to a diet higher in plant-based foods and lower in animal-based foods on the development of T2D, irrespective of general healthfulness of the specific plant-based and animal-based foods. With these results, we provide a different view on what a healthy diet may entail. However, we acknowledge that our plant-based diet included positive scoring for some components that are not necessarily healthy choices for prevention of T2D, or a healthy diet in general. Sugary beverages, for example, have been associated with adverse effects for T2D in other studies [22, 23].

To further clarify whether these less healthy plant foods contributed to the observed associations, we examined the associations between less healthy plant-based diet score with insulin resistance, and risk of prediabetes and T2D in our sensitivity analyses, and observed null associations; suggesting beneficial associations were mainly driven by higher intake of healthy plant-based food groups and lower intake of animal-based food groups. This emphasizes that it is important to also consider the quality of plant-based foods consumed, which has important public health implications. Furthermore, the estimates for the plant-based dietary index remained similar after excluding these plant-based beverages combined, or after excluding the less healthy plant-based foods combined, which indicated that our results were stable in diverse versions of plant-based diets, thus increased our confidence in the validity of the findings. We also observed that excluding each one of 23 components one by one at a time resulted in similar associations as observed for the total plant-based index, indicating that the associations were not mainly explained by any one specific food group, which supports the importance of recognizing overall plant-based diet. Finally, we extended our analyses to examine if adherence to a plant-based diet was independent of adherence to current Dutch dietary guidelines. In line with results from the large prospective cohort study in the US which examined if adherence to a plant-based diet was independent of general healthy dietary patterns that have been linked to prevention of T2D, such as the Mediterranean diet, the alternative Healthy Eating Index (aHEI), and the Dietary approaches to stop hypertension (DASH) diet [24–26]. We observed that associations of the plant-based dietary index with outcomes remained similar after additional adjustment for adherence to current Dutch dietary guidelines. This lends support to novelty of the plant-based dietary index.

Taken together, a more plant-based, less animal-based diet may help prevent the development of T2D. Still more important, a more plant-based diet, does not require a radical change in diet or a total elimination of meat or animal products but instead can be achieved in various ways, increasing the potential for population-wide health recommendations. For example, if a participant in our cohort would increase fruits intake from 95 to 200 g/day, increase vegetables intake from 100 to 260 g, and at the same time decrease red meat intake from 129 to 55 g/day, this would improve the plant-based dietary index by 10 units, which may decrease risk of T2D by 13%, assuming other covariates remain stable.

### Potential biological mechanisms

Several mechanisms behind the inverse associations could involve the intermediate conditions of T2D, such as obesity and inflammation, can offer explanations for the observed protection and T2D. On the one hand, a plant-based diet usually has more fiber, chlorogenic acids, certain amino acids, unsaturated fatty acids, and anti-oxidants. For example, vegetables and fruits are the main sources of fiber, anti-oxidants, and chlorogenic acids; nuts are rich in poly-unsaturated fatty acids; soy and beans are main sources of plant protein; whole grains are rich in fiber and plant protein; and coffee and tea are rich in anti-oxidants and phenol chlorogenic acid. These beneficial components may influence the development of T2D through impact on the potential intermediate conditions, such as obesity and inflammation. Fiber is known to lower gastric emptying and thereby glycemic responsiveness [27], and might improve inflammation [28, 29], and obesity [30]. Chlorogenic acids can improve inflammation, glucose tolerance and glucose levels, and improve increasing insulin secretion [31]. Soy protein contains high amounts of the amino acids arginine and glycine, which have been associated with a decrease in cholesterol levels [32]. High intake of unsaturated fatty acids has also been associated to lower inflammation and less obesity [28, 33]. Phenol chlorogenic acid was reported to reduce insulin resistance [34]. On the other hand, a plant-based diet, usually has less animal protein, saturated fatty acids, and heme iron. Animal protein is rich in branched-chain amino acids and aromatic amino acids and may impair glucose metabolisms and increase T2D risk [35–38]; animal protein is also rich in heme iron, which has been suggested to increase risk of cardio-metabolic diseases [39–41]. Higher saturated fatty acids have been suggested to be associated higher inflammation [33], higher risk of obesity [33] and T2D [42, 43]. Besides, other nutrients from processed red meat, such as sodium and nitrites, may increase risk of cardio-metabolic diseases [41]. More research is needed to explore whether the mechanisms also involve an effect of plant foods on gut microbiome. Finally, these different mechanisms may influence each other because of inter-relations between different food components. This also highlights the relevance of examining overall diets in additional to isolated food items, as this enables capturing of the combined effects of the potential pathways.

### Strengths and limitations

This study has several strengths. First, to our knowledge, we are the first to investigate the associations between plant-based diets with longitudinal insulin resistance and prediabetes, for which we had longitudinal data from long follow-up available. Studying these early risk stages help minimize reverse causation, understand how plant-based diet influences the development of T2D. Second, we observed that the potential beneficial effect of a more plant-based diet was independent of less healthy plant foods, such as sweets, sugary beverages and refined grains, emphasizing the importance of considering the quality of plant-based foods consumed. We also observed associations of the plant-based dietary score independent of overall adherence to dietary guidelines, indicating that the plant-based diet score may reflect more than only a healthful dietary pattern as reflected by current dietary guidelines. Other strengths also included the population-based nature of the study, the detailed and thorough data collected on the outcomes and the assessment of the extent to which diets were plant-based and animal based, based upon overall dietary intake patterns of the general population.

Nevertheless, there are several limitations we should consider. First, the assessment of a plant-based diet with this index has its limitations as several sometimes arbitrary decisions had to be made. A decision was, for example, to add up food items within categories based on the intake in grams per day. As a result, products that were high in water-content will have contributed less energy or nutrients compared to products containing less water in the same category. However, using grams per day reflects intake of foods as they are consumed and recommended [19]. Also, decisions had to be made for the categorization of foods and the number of categories. We chose categories reflecting those used in the Dutch dietary guidelines, which are based on similarities of the food items in (botanical) origin, nutrient composition, and nutrient density [18]; thereby reducing nutritional differences between food items within one category. Furthermore, in our main analyses, we treated all plant-based foods equally by giving all plant-based foods positive scores, and all animal-based foods equally by giving all animal-based foods reverse scores, irrespective of their nutrient-density or previous evidence for a role in T2D prevention and general health. For example, less healthy plant-based foods, such as sugary beverages and refined grains, were included as positive scores, although sugary beverages [23], and refined grains [44] have been linked to higher T2D risk; by contrast, healthy animal-based foods, such as dairy and fish, were included as reverse scores, although dairy [45] and fish [46] have been linked to lower T2D risk or mortality risk. That is because our study aimed to emphasize an overall plant-based diet including various increased plant-based foods consumption and decreased animal-based foods consumption, which would increase the potential for population-wide recommendation. However, in our sensitivity analyses, excluding any one of alcoholic beverages, sugary beverages, sweets, potatoes, refined grains, fish, and dairy did not substantially change our estimates.

In addition to the choices we had to make in the construction of the index, this study has some other limitations. First, dietary data were derived from self-reported diet measured with FFQs, making measurement-errors likely. However, because we used relative scores (quintiles) of intake and the FFQs were shown in several validation studies to adequately rank subjects according to intake [13–16], we do not expect these measurement-errors to have largely affected our results. Second, we did not have dietary data for many of the participants of the original cohort, which might have resulted in selection bias if associations of plant-based diets with T2D risk differed in those included and those not included in our current analyses. Third, we assumed stable diets over time. However, the estimates were similar after excluding the participants who were likely to change their diet during follow-up, such as participants with CHD, stroke, and cancers at baseline. Last, our results may be generalizable only to people of similar age and race.

## Conclusions

In this large population-based cohort, higher adherence to an overall plant-based diet is associated with lower longitudinal insulin resistance, and lower risk of prediabetes and T2D, indicating a protective role of diets high in plant-based foods and low in animal-based foods in the development to T2D beyond strict adherence to a vegetarian or vegan diet. These promising findings call for further exploration of overall plant-based dietary recommendations aimed at T2D prevention.

## Electronic supplementary material

Below is the link to the electronic supplementary material.

Supplementary material 1 (DOCX 68 kb)

Supplementary material 2 (DOC 89 kb)

## Abbreviations

T2D: Type 2 diabetes
HOMA-IR: Homeostatic model assessment of insulin resistance
BMI: Body mass index
CI: Confidence interval
HR: Hazard ratio
RS: Rotterdam Study
SD: Standard deviation
