# Supplementary material for: Plant versus animal based diets and insulin resistance, prediabetes and type 2 diabetes: the Rotterdam Study
Source: Eur J Epidemiol. 2018 Jun 8;33(9):883–93. doi: 10.1007/s10654-018-0414-8 (PMC6133017; doi:10.1007/s10654-018-0414-8)
Supplement: Supplementary file 1 — Supplementary material 1 (DOCX 68 kb) [file 10654_2018_414_MOESM1_ESM.docx]

**Online-only Supplemental Material**

**Supplemental Table 1 Food categories used for the plant-based diet index and examples of food items included in each of the food categories**

| Plant-based food categories | |
| --- | --- |
| *Fruits* | Apple, banana, pear, orange, strawberry, grapes, other fruits |
| *Vegetables* | Cauliflower, broccoli, spinach, carrots, onion, lettuce, tomato, cabbage, cooked vegetables |
| *Whole grains* | Whole grain bread, dark bread, rye bread, whole grain breakfast oats, whole grain pasta, brown rice |
| *Nuts* | Peanuts, walnuts, other nuts, peanut butter |
| *Legumes* | Legumes, tofu, soybeans, other soy products |
| *Potatoes* | Potatoes, fries |
| *Vegetable oils* | Olive oil, vegetable oils used for cooking, and all margarines |
| *Tea and coffee* | Black tea, green tea, herbal tea, coffee |
| *Sugary beverages* | Carbonated beverages with sugar, non-carbonated beverages with sugar, orange juice, fruit juice |
| *Refined grains* | Cornflakes, white bread, croissants, raisin bread, white pasta, white rice |
| *Sweets* | Sugar, cookies, cake, chocolate, candy-bars, honey, sweets, chocolate toppings, other sweet toppings |
| *Alcoholic beverages* | Red wine, white wine, beer, liquor, Dutch-eggnog |
| Animal-based food categories | |
| *Low-fat Yoghurt* | Skimmed yoghurt, semi-skimmed yoghurt, skimmed quark, buttermilk |
| *Full-fat Yoghurt* | Full-fat yoghurt, semi-skimmed quark, full quark |
| *Low-fat milk* | Skimmed milk, semi-skimmed milk, skimmed coffee creamer, semi-skimmed coffee creamer |
| *Full-fat milk* | Full-fat milk, cream, coffee-cream |
| *Cheese* | Full fat cheese, low fat cheese, cheese fondue, other cheese |
| *Fish* | Salmon, tuna, trout, herring, mussels, other fish |
| *Eggs* | Boiled eggs, fried eggs |
| *Animal fat* | Butter on bread, butter used for cooking, lard |
| *Desserts and sugary dairy* | Custard, cream, ice cream, mousse, cream, chocolate milk, fruit yoghurt, yoghurt drinks |
| *Unprocessed lean meat* | Chicken |
| *Processed meat and red meat* | Beef, pork, meatballs, sate, bacon, liver, processed meats |

**Online-only Supplemental Material**

**Supplemental Table 2 Baseline intake of 23 food categories of participants in quintiles of plant-based dietary index**

| Plant-based dietary index | Score≤43 | 43<Score≤47 | 47<Score ≤51 | 51<Score≤55 | Score˃55 |
| --- | --- | --- | --- | --- | --- |
|  | **n=1417** | **n=1311** | **n=1559** | **n=1226** | **n=1285** |
|  | **Median = 40** | **Median = 46** | **Median = 50** | **Median = 53** | **Median = 59** |
| Food intake (grams/day) |  |  |  |  |  |
| *Fruits* | 168.0 (83.4; 274.5) | 197.4 (104.0; 320) | 215.7 (115.2; 340.3) | 226.7 (127.3; 351.9) | 258.5 (161.1; 395.1) |
| *Vegetables* | 181.6 (128.0; 252.9) | 199.4 (143.9; 277.1) | 205.2 (146.4; 283.3) | 216.9 (156.4; 297.7) | 241.3 (180.4; 331.4) |
| *Whole grains* | 88.3 (46.6; 125.0) | 99.5 (50.0; 140.6) | 108.3 (63.0; 151.1) | 114.7 (67.6; 160.0) | 135.0 (80.0; 188.0) |
| *Legumes* | 0.0 (0.0; 8.9) | 0.0 (0.0; 16.9) | 4.1 (0.0; 18.0) | 7.8 (0.0; 24.0) | 13.5 (0.0; 35.6) |
| *Nuts* | 13.5 (0; 6.0) | 2.1 (0.0; 8.8) | 3.6 (0.0; 11.8) | 5.6 (0.4; 14.1) | 9.0 (2.7; 19.2) |
| *Vegetable oils* | 12.0 (3.3; 21.4) | 16.6 (7.2; 26.0) | 20.6 (10.4; 30.0) | 24.0 (13.3; 32.6) | 27.7 (18.1; 38.5) |
| *Tea and coffee* | 705.4 (500.0; 875.0) | 750.0 (525.0; 937.5) | 767.9 (597.1; 1000.0) | 812.5 (625.0; 1044.6) | 900.0 (705.4; 1125.0) |
| *Refined grains* | 37.7 (17.1; 76.8) | 50.0 (22.7; 97.6) | 50.6 (23.5; 101.3) | 60.0 (30.4; 115.6) | 61.2 (30.9; 122.2) |
| *Potato* | 83.6 (45.9; 122.0) | 88.2 (57.0; 131.0) | 97.9 (61.7; 142.5) | 108.3 (71.2; 163.1) | 126.0 (85.5; 178.1) |
| *Sweets* | 50.3 (26.6; 81.7) | 57.2 (32.6; 87.5) | 64.2 (38.2; 95.6) | 71.3 (43.5; 105.2) | 71.3 (43.5; 105.2) |
| *Sugary beverages* | 15.0 (0.0; 89.6) | 40.0 (0.0; 139.3) | 42.9 (0.0; 139.6) | 42.9 (0.0; 139.6) | 59.8 (1.2; 152.6) |
| *Alcoholic beverages* | 31.8 (2.5; 124.7) | 47.7 (3.6; 155.3) | 58.8 (4.9; 160.3) | 65.4 (8.4; 167.9) | 81.9 (14.2; 189.3) |
| *Low-fat yoghurt* | 82.3 (5.4; 192.9) | 64.1 (0.0; 166.1) | 60.0 (0.0; 164.5) | 53.6 (0.0; 162.0) | 32.1 (0.0; 149.6) |
| *Full-fat yoghurt* | 0.0 (0.0; 34.8) | 0.0 (0.0; 13.4) | 0.0 (0.0; 0.0) | 0.0 (0.0; 0.0) | 0.0 (0.0; 0.0) |
| *Low-fat milk* | 111.0 (1.9; 278.6) | 100.8 (0.88; 263.6) | 91.0 (0.0; 224.4) | 59.0 (0; 224.4) | 48.0 (0.0; 196.5) |
| *Full-fat milk* | 0.0 (0.0; 7.0) | 0.0 (0.0; 0.0) | 0.0 (0.0; 0.0) | 0.0 (0.0; 0.0) | 0.0 (0.0; 0.0) |
| *Cheese* | 32.9 (21.3; 47.1) | 32.6 (20.3; 50.0) | 30.3 (20.0; 46.6) | 28.4 (18.2; 44.6) | 29.9 (17.8; 47.0) |
| *Fish* | 21.4 (7.1; 33.8) | 18.9 (5.9; 33.0) | 14.6 (4.2; 30.2) | 14.4 (2.4; 28.6) | 11.0 (0.0; 25.9) |
| *Eggs* | 14.3 (8.9; 21.4) | 14.3 (7.1; 21.4) | 14.3 (7.1; 17.9) | 14.3 (7.1; 17.1) | 10.7 (7.1; 17.1) |
| *Animal fat* | 0.7 (0.0; 12.0) | 0.0 (0.0; 2.3) | 0.0 (0.0; 0.0) | 0.0 (0.0; 0.0) | 0.0 (0.0; 0.0) |
| *Desserts/dairy with sugars* | 21.4 (1.5; 63.9) | 18.4 (0.4; 60.5) | 14.9 (0.0; 59.6) | 10.2 (0.0; 48.1) | 6.4 (0.0; 35.8) |
| *Unprocessed lean meat* | 14.3 (6.9; 21.4) | 14.3 (7.1; 21.4) | 11.4 (4.3; 18.6) | 10.7 (4.3; 17.8) | 7.6 (0.0; 14.9) |
| *Processed meat / red meat* | 93.2 (65.4; 127.5) | 89.3 (63.4; 127.5) | 86.9 (60.0; 118.0) | 85.5 (60.4; 117.9) | 80.0 (52.5; 112.3) |

Variables expressed as median (IQR) because of their skewed distributions.

**Online-only Supplemental Material**

**Supplemental Table 3 Baseline characteristics of participants in original and multiple imputed dataset**

| Characteristics | Original data  Mean (SD) or valid % | After imputation Mean (SD) or % |
| --- | --- | --- |
| Age (years) | 62.0 (7.8) | NI |
| *Missing (%)* | *−* | *−* |
| Gender (% male) | 41.3 % | NI |
| *Missing (%)* | − | − |
| BMI (kg/m2) | 26.6 (3.9) | 26.6 (3.9) |
| *Missing (%)* | 1.3 % | − |
| Smoking status (%) |  |  |
| *Never* | 32.2 % | 32.2 % |
| *Ever* | 45.1 % | 45.1 % |
| *Current* | 22.7 % | 22.7 % |
| *Missing (%)* | 0.5 % | − |
| Physical activity^1^ (MET-hours/week) |  |  |
| *RS-III (assessed with LASA Questionnaire, n=2194)* | 58.4 (55.8) | 58.4 (55.8) |
| *RS-I and RS-II (assessed with Zutphen Questionnaire, n=4393)* | 86.7 (44.7) | 86.7 (44.7) |
| *Missing (%)* | 3.9 % | − |
| Hypertension (%) | 42.3 % | 42.3 % |
| *Missing (%)* | 0.9 % | − |
| Hypercholesterolemia (%) | 45.6 % | 45.4 % |
| *Missing (%)* | 1.6 % | − |
| Family history of type 2 diabetes (%) | 10.8 % | NI |
| *Missing (%)* | − | − |
| Education level (%) |  |  |
| *Primary* | 11.8 % | 11.8 % |
| *Lower* | 40.9 % | 40.9 % |
| *Intermediate* | 29.0 % | 29.0 % |
| *Higher* | 18.3 % | 18.3 % |
| *Missing (%)* | 0.6 % | − |
| Current food supplement use (%) | 16.5 % | 16.5 % |
| *Missing (%)* | 0.3 % | − |
| Total energy intake (kcal/day) | 2134 (615) | NI |
| *Missing (%)* | − | − |
| Food category intake^2^ (grams/day) |  |  |
| *Fruits* | 212.2 (115.5; 332.3) | NI |
| *Vegetables* | 209.1 (147.9; 286.87 | NI |
| *Whole grains* | 105.7 (61.3; 152.5) | NI |
| *Nuts* | 3.9 (0.0; 12.0) | NI |
| *Legumes* | 4.1 (0.0; 19.4) | NI |
| *Potatoes* | 99.7 (61.4; 148.2) | NI |
| *Vegetable oils* | 19.7 (9.2; 30.0) | NI |
| *Tea and coffee* | 758.9 (580.4; 1000) | NI |
| *Sugary beverages* | 46.3 (0.0; 139.6) | NI |
| *Refined grains* | 50.7 (23.9; 102.1) | NI |
| *Sweets* | 63.8 (37.1; 97.4) | NI |
| *Alcoholic beverages* | 56.4 (4.9; 159.8) | NI |
| *Low-fat milk* | 82.3 (0.0; 232.3) | NI |
| *Full-fat milk* | 0.0 (0.0; 0.0) | NI |
| *Low-fat yoghurt* | 56.1 (0.0; 164.6) | NI |
| *Full-fat yoghurt* | 0.0 (0.0; 4.9) | NI |
| *Cheese* | 30.8 (20; 47.1) | NI |
| *Unprocessed lean* | 10.7 (4.3; 18.1) | NI |
| *Fish* | 15.9 (3.9; 30.7) | NI |
| *Eggs* | 14.3 (7.1; 19.6) | NI |
| *Animal fat* | 0.0 (0.0; 0.9) | NI |
| *Desserts/dairy with sugars* | 14.1 (0.0; 54.6) | NI |
| *Processed meat / red meat* | 86.8 (60.4; 118.9) | NI |
| Plant-based dietary index (score) | 49.3 (7.1) | NI |
| Plant-based dietary index: a higher score indicates a higher adherence to a plant-based diet (theoretical range from 0 to 92).  Values shown are un-imputed; imputation was performed on z-scores of physical activity.  Variables expressed as median (IQR) because of their skewed distributions.  Abbreviations: MET, metabolic equivalent of task; NI, not imputed; SD, standard deviation. | | |

**Online-only Supplemental Material**

**Supplemental Table 4 Non-response analyses**

| Covariates | Participants without valid dietary data n=5225 | Participants with valid dietary data  n=9701 | P |
| --- | --- | --- | --- |
|  | **Mean (SD) or %** | **Mean (SD) or %** | **T-test or X^2^ test** |
| Age (years) | 64.9 (12.7) | 62.0 (7.8) | P<0.05 |
| Sex (%) |  |  |  |
| *Female* | 59.0% | 41.8% | P<0.05 |
| *Male* | 38.8% | 58.0% |  |
| BMI (kg/m2) | 27.0 (4.4) | 26.6 (3.9) | P<0.05 |
| Physical activity (MET-hours/week) |  |  | P<0.05 |
| *RS-I and RS-II (assessed with Zutphen Questionnaire)* | 72.4 (42.5) | 83.5 (44.6) | P<0.05 |
| *RS-III (assessed with LASA Questionnaire)* | 65.3(43.5) | 58.2 (59.3) |  |
| Education level (%) |  |  | p>0.05 |
| *Primary* | 25.0% | 11.8% |  |
| *Lower* | 37.2% | 40.9% |  |
| *Intermediate* | 24.4% | 29.0% |  |
| *Higher* | 13.3% | 18.4% |  |
| Smoking status (%) |  |  |  |
| *Never* | 35% | 32.2% | p>0.05 |
| *Ever* | 39% | 45.06% |  |
| *Current* | 25.6% | 22.7% |  |
| Current food supplement use (%) |  |  |  |
| *Yes* | 16.9% | 16.5% | p>0.05 |
| *No* | 83.1% | 83.2% |  |
| Family history of diabetes (%) |  |  |  |
| *Yes* | 9.0% | 10.8% | p>0.05 |
| *No* | 39.8% | 45.8% |  |
| *Unknown* | 51.3% | 43.4% |  |
| Covariates | **Participants not included in analyses**  **n=8128** | **Included participants in analyses**  **n=6798** | **P** |
|  | **Mean (SD) or %** | **Mean (SD) or %** | **T-test or X2 test** |
| Age (years) | 69.3 (11.4) | 62.0 (7.8) | P<0.05 |
| Sex (%) |  |  |  |
| *Female* | 59.5% | 57% | p>0.05 |
| *Male* | 40.1% | 41.3 % |  |
| BMI (kg/m2) | 27.1 (4.3) | 26.6 (3.9) | P<0.05 |
| Physical activity (MET-hours/week) |  |  |  |
| *RS-I and RS-II (assessed with Zutphen Questionnaire)* | 72.1 (42.5) | 86.7 (44.7) | P<0.05 |
| *RS-III (assessed with LASA Questionnaire)* | 61.6 (79.9) | 58.4 (55.8) |  |
| Education level (%) |  |  |  |
| *Primary* | 23.6% | 11.8 % | p>0.05 |
| *Lower* | 37.0% | 40.9 % |  |
| *Intermediate* | 23.6% | 29.0 % |  |
| *Higher* | 11.1% | 18.3 % |  |
| Smoking status (%) |  |  |  |
| *Never* | 32.5% | 32.2 % | p>0.05 |
| *Ever* | 38.4% | 45.1 % |  |
| *Current* | 24.3% | 22.7 % |  |
| Current food supplement use (%) |  |  |  |
| *Yes* | 14.6% | 16.5% | P<0.05 |
| *No* | 84.6% | 83.5% |  |
| Family history of diabetes (%) |  |  |  |
| *Yes* | 13.9% | 45.8% | p>0.05 |
| *No* | 49.1% | 10.8% |  |
| *Unknown* | 36.9% | 43.4% |  |

t-test was performed for continuous variables, and X^2^ was performed for categorical variable

**Online-only Supplemental Material**

**Supplemental Table 5 Associations of the plant-based dietary index with longitudinal insulin resistance (HOMA-IR) for the three sub-cohorts separately**

|  | β for HOMA-IR (95% CI) | | |
| --- | --- | --- | --- |
|  | **RS-I (n=2892)** | **RS-II (n=1389)** | **RS-III (n=2233)** |
| Model 1 | -0.09 (-0.10; -0.08)*** | -0.07 (-0.11; -0.03)*** | -0.11 (-0.14; -0.07)*** |
| Model 2 | -0.09 (-0.10; -0.08)*** | -0.06 (-0.10; -0.02)** | -0.10 (-0.13; -0.07)*** |
| Model 3 | -0.05 (-0.07; -0.03)* | -0.01 (-0.05; 0.02) | -0.06 (-0.09; -0.03)*** |
| Effect estimates are βs for ln-transformed HOMA-IR per 10 units higher score on the plant-based dietary index and are based on pooled results of the imputed dataset.  Model 1 is adjusted for energy intake (kcal), sex (male or female), age (years), and time (years) of repeated measurements of longitudinal insulin resistance.  Model 2 is additionally adjusted for education (primary education, lower/intermediate general education or lower vocational education, intermediate vocational education or higher general education, higher vocational education or university), smoking status (never, ever, current), family history of diabetes (yes, no, or unknown); physical activity (z-score of MET-hours/week); and food supplement use (yes or no).  Model 3 is additionally adjusted for BMI (kg/m2).  *p<0.05; **p<0.01; ***p<0.001  Abbreviations: CI, confidence interval; HOMA-IR, homeostasis model assessment for insulin resistance; MET, metabolic equivalent of task; RS, Rotterdam-Study. | | | |

**Online-only Supplemental Material**

**Supplemental Table 6 Associations of the plant-based dietary index with incidence of prediabetes for the three sub-cohorts separately**

|  | HR (95% CI) for prediabetes | | |
| --- | --- | --- | --- |
|  | **RS-I (n=2492)** | **RS-II (n=1151)** | **RS-III (n=2125)** |
| Model 1 | 0.93 (0.82; 1.05) | 0.94 (0.78; 1.14) | 0.65 (0.51; 0.84)*** |
| Model 2 | 0.94 (0.83; 1.06) | 0.94 (0.78; 1.14) | 0.66 (0.52; 0.85)** |
| Model 3 | 0.96 (0.85; 1.09) | 1.00 (0.83; 1.21) | 0.70 (0.54; 0.90)** |
| Effect estimates are HRs (95% CIs) for incidence of prediabetes per 10 units higher score on the plant-based dietary index and are based on pooled results of the imputed dataset.  Model 1 is adjusted for energy intake (kcal), sex (male or female), and age (years),.  Model 2 is additionally adjusted for education (primary education, lower/intermediate general education or lower vocational education, intermediate vocational education or higher general education, higher vocational education or university), smoking status (never, ever, current), family history of diabetes (yes, no, or unknown); physical activity (z-score of MET-hours/week); and food supplement use (yes or no).  Model 3 is additionally adjusted for BMI (kg/m2).  *p<0.05; **p<0.01; ***p<0.001  Abbreviations: BMI, body mass index; CI, confidence interval; HR, hazard ratio; MET, metabolic equivalent of task; RS, Rotterdam-Study. | | | |

**Online-only Supplemental Material**

**Supplemental Table 7 Associations of the plant-based dietary index with incidence of type 2 diabetes for the three sub-cohorts separately**

|  | HR (95% CI) for type 2 diabetes | | |
| --- | --- | --- | --- |
|  | **RS-I (n=2975)** | **RS-II (n=1411)** | **RS-III (n=2384)** |
| Model 1 | 0.85 (0.73; 0.98)* | 0.82 (0.65; 1.02) | 0.74 (0.54; 1.02) |
| Model 2 | 0.86 (0.74; 0.997)* | 0.86 (0.69; 1.07) | 0.75 (0.54; 1.04) |
| Model 3 | 0.91 (0.78; 1.05) | 0.93 (0.74; 1.16) | 0.80 (0.58; 1.12) |
| Effect estimates are HRs (95% CIs) for incidence of type 2 diabetes per 10 units higher score on the plant-based dietary index and are based on pooled results of the imputed dataset.  Model 1 is adjusted for energy intake (kcal), sex (male or female), and age (years).  Model 2 is additionally adjusted for education (primary education, lower/intermediate general education or lower vocational education, intermediate vocational education or higher general education, higher vocational education or university), smoking status (never, ever, current), family history of diabetes (yes, no, or unknown); physical activity (z-score of MET-hours/week); and food supplement use (yes or no).  Model 3 is additionally adjusted for BMI (kg/m2).  *p<0.05; **p<0.01; ***p<0.001  Abbreviations: BMI, body mass index; CI, confidence interval; HR, hazard ratio; MET, metabolic equivalent of task; RS, Rotterdam-Study. | | | |

**Online-only Supplemental Material**

**Supplemental Table 8 Associations of the plant-based dietary index with longitudinal insulin resistance (HOMA-IR), risk of prediabetes and type 2 diabetes (T2D) after excluding each one of 23 components one by one at a time, and additionally adjusting for the excluded one**

| Plant-based dietary index with 22 components instead of 23 components | β (95% CI) for HOMA-IR | HR (95% CI) for Prediabetes risk | HR (95% CI) for T2D risk |
| --- | --- | --- | --- |
|  | **n=6514** | **n=5768** | **n=6770** |
| Excluding fruits | -0.08 (-0.10; -0.07) *** | 0.89 (0.81; 0.98) * | 0.82 (0.73; 0.92) ** |
| Excluding vegetables | -0.09 (-0.10; -0.09) *** | 0.89 (0.81; 0.98) * | 0.81 (0.72; 0.92) ** |
| Excluding whole grains | -0.09 (-0.10; -0.09) *** | 0.89 (0.81; 0.98) * | 0.81 (0.73; 0.92) ** |
| Excluding nuts | -0.07 (-0.09; -0.06) *** | 0.91 (0.81; 1.00) | 0.84 (0.76; 0.95) ** |
| Excluding legumes | -0.08 (-0.10; -0.07) *** | 0.90 (0.82; 0.99) * | 0.83 (0.74; 0.92) ** |
| Excluding vegetable oils | -0.08 (-0.10; -0.07) *** | 0.90 (0.82; 0.99) * | 0.82 (0.73; 0.92) ** |
| Excluding tea and coffee | -0.07 (-0.09; -0.06) *** | 0.91 (0.83; 0.99) * | 0.84 (0.75; 0.95) ** |
| Excluding potatoes | -0.09 (-0.10; -0.09) *** | 0.89 (0.81; 0.98) * | 0.82 (0.73; 0.92) ** |
| Excluding sugary beverages | -0.09 (-0.10; -0.08) *** | 0.89 (0.81; 0.98) * | 0.82 (0.72; 0.92) ** |
| Excluding refined grains | -0.09 (-0.10; -0.08) *** | 0.89 (0.81; 0.98) * | 0.82 (0.73; 0.92) ** |
| Excluding sweets | -0.08 (-0.10; -0.08) *** | 0.90 (0.82; 0.99) * | 0.81 (0.73; 0.92) ** |
| Excluding alcoholic beverages | -0.08 (-0.10; -0.06) *** | 0.89 (0.82; 0.98) * | 0.83 (0.71; 0.95) ** |
| Excluding red and processed meat | -0.07 (-0.08; -0.07) *** | 0.93 (0.84; 0.99) * | 0.84 (0.76; 0.95) ** |
| Excluding unprocessed lean meat | -0.07(-0.08; -0.07) *** | 0.90 (0.82; 0.99) * | 0.84 (0.76; 0.95) ** |
| Excluding fish | -0.08 (-0.10; -0.07) *** | 0.90 (0.81; 0.99) * | 0.84 (0.74; 0.94) ** |
| Excluding eggs | -0.09 (-0.10; -0.08) *** | 0.89 (0.80; 0.98) * | 0.82 (0.73; 0.92) ** |
| Excluding animal fat | -0.08 (-0.10; -0.08) *** | 0.89 (0.79; 0.99) * | 0.83 (0.70; 0.95) ** |
| Excluding cheese | -0.08 (-0.10; -0.07) *** | 0.91 (0.82; 0.99) * | 0.84 (0.75; 0.94) ** |
| Excluding low-fat milk | -0.08 (-0.10; -0.06) *** | 0.86 (0.79; 0.95) * | 0.81 (0.72; 0.92) ** |
| Excluding full-fat milk | -0.08 (-0.10; -0.07) *** | 0.90 (0.82; 0.99) * | 0.83 (0.72; 0.93) ** |
| Excluding low-fat yoghurt | -0.08 (-0.10; -0.07) *** | 0.89 (0.81; 0.98) * | 0.82 (0.74; 0.92) ** |
| Excluding full-fat yoghurt | -0.09 (-0.10; -0.09) *** | 0.86 (0.78; 0.94) * | 0.80 (0.70; 0.90) ** |
| Excluding desserts/dairy with sugars | -0.08 (-0.10; -0.08) *** | 0.90 (0.81; 0.99) * | 0.83 (0.71; 0.94) ** |

Effect estimates are regression coefficients (β) for ln HOMA-IR or hazard ratios (HRs) for incidence of prediabetes or type 2 diabetes with their 95%-confidence intervals (95%CIs), per 10 units higher score on the plant-based dietary index by excluding one of 23 foods at a time and additionally adjusting for the excluded food group. Estimates are adjusted for total energy, age, sex, RS sub-cohort, education, smoking status, family history diabetes, physical activity, and food supplement use (only for the HOMA analyses additionally for the time measurements of longitudinal HOMA), based on pooled results of imputed data. *p<0.05; **p<0.01; ***p<0.001
